# A Five-Year Retrospective Study from a Single Center on the Location, Presentation, Diagnosis, and Management of 110 Patients with Aneurysms of the Femoral and Popliteal Arteries of the Lower Limb

**DOI:** 10.3390/jcm13154323

**Published:** 2024-07-24

**Authors:** Michał Serafin, Dorota Łyko-Morawska, Julia Szostek, Dariusz Stańczyk, Magdalena Mąka, Iga Kania, Wacław Kuczmik

**Affiliations:** Department of General Surgery, Vascular Surgery, Angiology and Phlebology, Faculty of Medical Sciences in Katowice, Medical University of Silesia, 14 Medyków Street, 45-47 Ziołowa Street, 40-635 Katowice, Poland; dorota.lyko@gmail.com (D.Ł.-M.); s81394@365.sum.edu.pl (J.S.); darekstanczyk@interia.pl (D.S.); s81151@365.sum.edu.pl (M.M.); s80989@365.sum.edu.pl (I.K.); wkuczmik@interia.pl (W.K.)

**Keywords:** lower limb, aneurysm, pseudoaneurysm, endovascular procedure, surgery

## Abstract

**Background:** Peripheral aneurysms, although known about for centuries, are challenging to monitor due to their asymptomatic nature. Advanced imaging has improved detection, which is crucial for preventing emergent complications. This five-year retrospective study from a single center aimed to evaluate the location, presentation, diagnosis, and management of 110 patients with aneurysms of the femoral and popliteal arteries of the lower limb. **Materials and methods:** The study included 71 true aneurysms and 39 pseudoaneurysms patients treated between 2018–2023. Treatment methods were based on aneurysm size, atherosclerosis severity, and operation risk. The study assessed patient demographics, surgical details, postoperative complications, and aneurysm characteristics. **Results:** Acute limb ischemia was more prevalent in true aneurysms (25.4% vs. 7.7%; *p* = 0.02). Aneurysmectomy was performed more frequently in pseudoaneurysms (87.2% vs. 54.9%; *p* < 0.001), while endovascular treatment and surgical bypass were more common in true aneurysms (Endovascular: 22.5% vs. 2.6%; *p* = 0.01; bypass: 21.1% vs. 0%; *p* < 0.001). Early postoperative complications occurred in 22.7% of patients. The 12-month freedom from reoperations (73.7% vs. 87%; *p* = 0.07), amputations (97.7% vs. 93.8%; *p* = 0.2), and graft stenosis (78.7% vs. 86.87%; *p* = 0.06) showed no significant differences between groups. **Conclusions:** Lower limb aneurysms often present with non-specific symptoms, leading to late diagnosis and life-threatening complications. Both open and endovascular treatments are feasible, though more research is needed for pseudoaneurysms. Vigilant follow-up is crucial due to potential adverse events, though overall mortality and morbidity remain low.

## 1. Introduction

The first peripheral artery aneurysm (PA) was described over 4000 years ago in the Ebers Papyrus (ca. 2000 BC) [1]. Today, its incidence is difficult to define due to its asymptomatic nature. However, improved imaging techniques and better healthcare availability have increased detectability [2]. The two-thirds of all PAs are lower limb aneurysms [3,4]. PAs are classified into true aneurysms, a vessel dilatation involving all arterial wall layers, and pseudoaneurysms, defined as a dilation caused by a hematoma contained only by the tunica adventitia or a pseudo-fibrous capsule [3]. The incidence varies depending on the location and type of aneurysm.

The etiology of lower limb true aneurysms, as well as general true aneurysms, is unknown. Molecular studies suggest that a combination of genetic defects and inflammatory processes is responsible [5,6]. Pseudoaneurysms are related to traumatic injury of the vessel wall, leading to blood entering the surrounding tissue and forming a pulsatile hematoma [3,7].

In up to 40% of cases, lower limb aneurysms are clinically silent [3,5]. Symptoms can be easily overlooked [5,8]. The most common symptoms include lower limb pain and a palpable mass, while up to 77% of symptomatic patients may present life-threatening complications such as chronic thrombosis with intermittent claudication, acute limb ischemia due to acute thrombosis, and aneurysm rupture [3,8,9].

Duplex ultrasonography (USG) should be the preferred method for screening and detecting femoral and popliteal aneurysms [10]. Computed tomography angiography (CTA) is the most effective diagnostic method for evaluating lower limb aneurysms before surgical intervention [4,7].

Interventions are primarily indicated for symptomatic lower limb aneurysms. Current guidelines recommend repairing asymptomatic popliteal artery aneurysms (PAAs) ≥20 mm in diameter to mitigate the risk of thromboembolic events and limb loss. For patients at higher clinical risk, repair may be delayed until the aneurysm reaches ≥30 mm. For PAAs <20 mm, repair should be considered if there is a thrombus, clinical suspicion of embolism, or poor distal runoff on imaging. Open PAA repair is advised for asymptomatic patients with a life expectancy of at least 5 years and an adequate saphenous vein. For those with reduced life expectancy, endovascular repair should be considered [11]. Symptomatic common femoral artery aneurysms or asymptomatic ones over 2.5 cm warrant surgical intervention [12]. Treatment options include minimally invasive methods like ultrasound-guided compression or thrombin injection, and classical surgical procedures [13]. Endovascular treatment is also increasingly used for lower limb aneurysms [13]. Given the mobility of the lower limb, stent grafts that are flexible and resistant to kinking should be used [12,14,15].

Given the diversity of lower limb aneurysms and their treatment options, appropriate treatment should be tailored to each case, considering the aneurysm’s etiology, location, and diameter, as well as the patient’s clinical status and postoperative quality of life [5,8].

The primary objective of this study is to clinically characterize lower limb aneurysms and analyze the perioperative, short-term, and long-term outcomes of surgical treatment based on data from the Department of General Surgery, Vascular Surgery, Angiology, and Phlebology at the Medical University of Silesia in Katowice, Poland. This five-year retrospective study evaluates the location, presentation, diagnosis, and management of 110 patients with lower limb artery aneurysms.

## 2. Materials and Methods

### 2.1. Study Design and Population

The retrospective study included all patients from the Department of General Surgery, Vascular Surgery, Angiology, and Phlebology at the Medical University of Silesia in Katowice, Poland, who were treated with open or endovascular methods for a femoral (common, deep, superficial) or popliteal artery aneurysm from January 2018 to December 2023. We individually reviewed the medical records from the electronic system for all treatments of lower limb aneurysms and analyzed only those with primary open or endovascular procedures. Patients who received interventions for recurrent aneurysms were excluded from further analysis.

**Figure 1:**
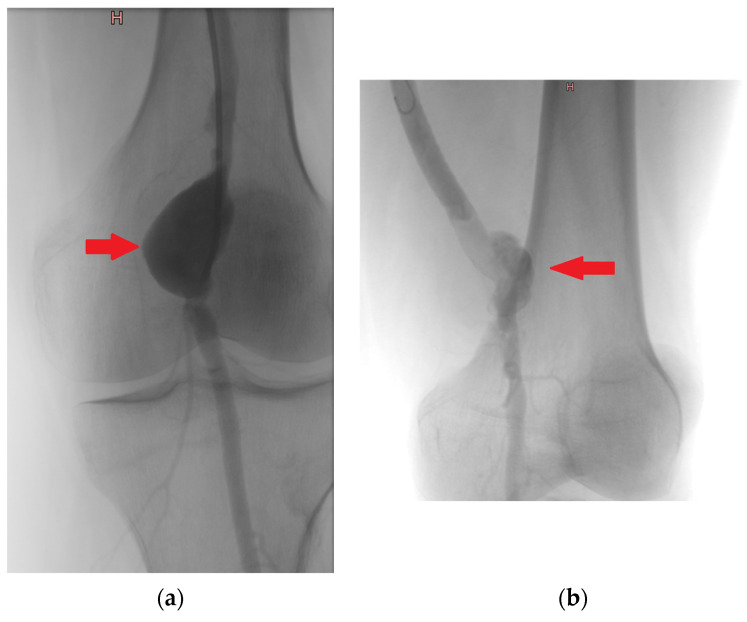
Intraoperative angiography—true aneurysm of popliteal artery (**a**), pseudoaneurysm of popliteal artery (**b**). Note: (**a**) (red arrow)—a smooth, rounded expansion of the artery involving the entire circumference of the vessel, indicating that all three layers of the arterial wall (intima, media, and adventitia) are involved. (**b**) (red arrow) an irregular dilation of the artery that is contained by periarterial connective tissue.

The study group consisted of 110 adult patients (95, 86.4% men; 15, 13.6% women), aged 15–98 years old (67.5 IQR 10) and was divided into two subgroups according to the type of aneurysm: true aneurysm (n = 71; 64.5%) and pseudoaneurysm (n = 39; 35.5%; Figure 1).

### 2.2. Diagnostic Criteria

**Figure 2:**
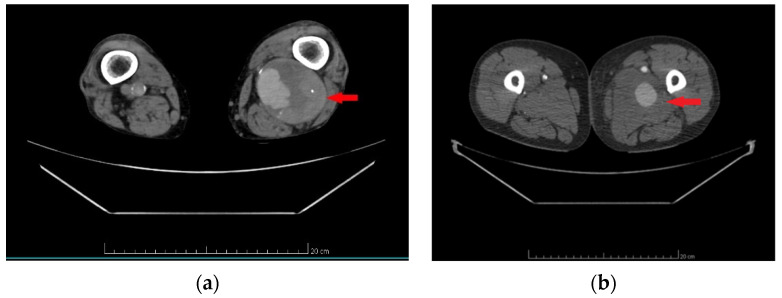
Computed tomography angiography—true aneurysm of popliteal artery (**a**), pseudoaneurysm of common femoral artery (**b**). Note: (**a**) (red arrow)—round, sharply demarcated from the surrounding tissues dilatation with the involvement of the intima, media, and adventitia of the popliteal artery. (**b**) (red arrow)—poorly demarcated common femoral artery dilations contained by periarterial connective tissue.

True aneurysms were diagnosed using computed tomography angiography (CTA), following the 2022 clinical practice guidelines from the Society for Vascular Surgery on popliteal aneurysms and the guidelines for the Management of Patients with Peripheral Arterial Disease. The diagnostic criteria for true aneurysms included a focal, isolated dilation of the vessel exceeding 1.5 times the diameter of the normal, healthy segment of the artery, with the aneurysm wall encompassing all three layers of the arterial wall: intima, media, and adventitia [11,16]. An example angiographic image showing the characteristic dilation of the vessel with all three arterial wall layers involved can be seen in Figure 2a.

Pseudoaneurysms were diagnosed using CTA, in accordance with the European Society for Vascular Surgery clinical practice guidelines from 2011, updated in 2019. The diagnostic criteria for pseudoaneurysms included a dilation or outpouching of the artery, consisting of one or two layers of the vessel wall, contained only by periarterial connective tissue [17,18]. An example CTA image showing the outpouching of the artery surrounded by periarterial connective tissue defining the boundary is provided in Figure 2b.

The patients with lower limb aneurysm were considered for invasive treatment in case of the symptomatic aneurysm/pseudoaneurysm or in case of asymptomatic common femoral artery aneurysm/pseudoaneurysm that exceeded 25 mm or asymptomatic popliteal artery aneurysm/pseudoaneurysm exceeding 20 mm [11,16,17,18].

### 2.3. Methods of Treatment

All patients before surgical treatment were evaluated by a multispecialist team of vascular surgeons and radiologists who treated the patients and determined their qualification for a specific procedure based on CTA.

The inclusion criteria for aneurysmectomy/surgical bypass/endovascular treatment/Aneurysmectomy with surgical bypass are detailed below.

Aneurysmectomy was performed in cases of aneurysm/pseudoaneurysm with mild atherosclerosis of the artery before and after the aneurysm sac, accompanied by good blood flow. This procedure was considered when the vessel exhibited limited atherosclerotic changes, ensuring that the integrity of the artery could be maintained and optimal blood flow could be preserved.

Surgical bypass was performed in cases of aneurysm with severe atherosclerosis of the artery before and after the aneurysm sac, accompanied by reduced blood flow. This procedure was chosen when the arterial segments adjacent to the aneurysm were significantly affected by atherosclerotic plaque, compromising blood circulation. The procedure aimed to reroute blood flow around the obstructed sections and aneurysm sac, restoring adequate perfusion to the lower limb and preventing the growth of the aneurysm.

Aneurysmectomy with surgical bypass was performed in cases of aneurysm compression of the vein and/or nerve or anastomotic pseudoaneurysms with severe atherosclerosis of the artery before and after the aneurysm sac, accompanied by reduced blood flow. This combined approach was necessary when the aneurysm or pseudoaneurysm was sizable, and the surrounding arterial segments were heavily affected by atherosclerotic changes, leading to compromised blood circulation. Additionally, symptomatic nerve or vein compression mandated surgical repair with aneurysm resection. This intervention was critical to relieve pressure on the affected nerves or veins and restore normal function.

In addition, according to clinical practice guidelines [11], during open procedures, patients with acute limb ischemia underwent simultaneous surgical thrombectomy. In cases of chronic occlusive disease, patients underwent simultaneous endarterectomy. This approach allowed us to minimize the radiation exposure received by the patient while simultaneously restoring proper circulation to the lower limb.

Endovascular treatment was performed in high-risk patients with contraindications for open surgical treatment. This minimally invasive procedure was chosen for individuals who were deemed unsuitable for traditional open surgery due to factors such as advanced age, comorbidities, or other medical conditions that increased the risk of surgical complications. Endovascular treatment allowed for effective management of the aneurysm while minimizing the overall risk to the patient.

In addition, according to clinical practice guidelines [11], during the endovascular procedures, patients with acute limb ischemia underwent simultaneous mechanical thrombectomy, while in the case of chronic occlusive disease, patients underwent simultaneous percutaneous angioplasty with/without stenting. This approach allowed us to quickly restore proper circulation to the lower limb.

During aneurysmectomy and surgical bypass, the SilverGraf (B. Braun Melsungen AG, Berlin, Germany) or Gelsoft™ Plus Vascular Prosthesis (Terumo Aortic, Inchinnan, UK) was used. The type of vascular prosthesis was chosen by the main vascular surgeon performing the procedure.

The GORE VIABAHN VBX Balloon Expandable Endoprosthesis (W. L. Gore & Associates, Flagstaff, AZ, USA) was used in endovascular treatment.

### 2.4. Definitions

Early postoperative complications were defined as the occurrence of complications during a patient’s hospitalization or during 30 days after the surgical procedure.

Early reoperation was defined as the performance of additional procedures due to the occurrence of early postoperative complications during a patient’s hospitalization or during 30 days after the surgical procedure.

Late postoperative complications were defined as the occurrence of complications >30 days after the surgical procedure.

Late reoperation was defined as the performance of additional procedures due to the occurrence of late postoperative complications >30 days after the surgical procedure.

The total duration of hospitalization was defined from the day of admission to the day of discharge from the hospital. Meanwhile, the duration of postoperative hospitalization was defined as the interval between the day of surgery and the day of discharge.

Follow-up was defined as the patient’s last registered visit to the ward or clinic.

Overall survival (OS) was measured from the date of the surgical procedure to either the date of death or the date of the last contact in the ward or clinic.

Graft stenosis was delineated as a constriction of the graft surpassing 75% and manifested by a >3.5-fold increase in peak systolic velocity, as ascertained via duplex ultrasound or angiographic evaluation.

Amputations were defined as a major below-knee, above-knee, or femoral amputation.

Reintervention was warranted for grafts demonstrating symptomatic stenosis and for asymptomatic grafts deemed at risk, defined as those exhibiting a peak systolic velocity ratio >3.5 to 4 or displaying low graft velocities <30 cm/s.

Reoperation-amputation-stenosis (RAS events) was defined as a variable denoted by the occurrence of reoperation, amputation, or graft stenosis at any time after the primary procedure.

### 2.5. Analyzed Data

Parameters such as patients’ general characteristics (i.e., age, gender, comorbidities, clinical symptoms), the type and the duration of surgery, incidence of postoperative complications, reoperations, mortality, duration of hospitalization as well as the aneurysm localization, diameter, and follow-up were analyzed in the study.

The patients’ general characteristics (i.e., age, gender, comorbidities, clinical symptoms), as well as details about the incidence of postoperative complications (including graft stenosis) and reoperations, were obtained from the patient’s medical history from our department.

Details about the localization of the aneurysms, as well as the duration and type of the procedure, were collected from the description of the patient’s surgery.

Aneurysm diameter was obtained from each patient’s CTA.

Follow-up data such as late complications (including graft stenosis), late reoperations, and mortality were obtained from the patient’s medical history from the surgical clinic and/or from the department.

### 2.6. Statistical Analysis

The statistical analysis was performed using Statistica^®^ (Tulsa, OK, USA, 2013) software version 13.3 (StatSoft). Absolute values and percentages were used to present qualitative variables. Ranges, means, and standard deviations or medians with interquartile ranges were applied for quantitative variables. The Shapiro–Wilk test was used to determine statistical distribution in the analyzed patients. Between-group comparisons were performed for two types of aneurysm (true aneurysm and pseudoaneurysm) in terms of demographics, peri- and postoperative parameters, as well as follow-up parameters. Analysis was performed using the chi-square test, Fisher’s exact test, or the Mann–Whitney U test. The predictive factors of early postoperative complications were calculated using univariate logistic regression analysis. Overall survival analysis, freedom from reoperations, amputations, graft stenosis, and RAS events were performed using the Kaplan–Meier estimator. The log-rank test was used to compare the overall survival analysis, freedom from reoperations, amputations, graft stenosis, and RAS events between the true aneurysm and pseudoaneurysm groups. The analysis of prognostic factors for the overall survival analysis, freedom from reoperations, amputations, graft stenosis, and RAS events was performed using the univariate Cox proportional hazards regression model and included the patient’s age, gender, presence of arterial hypertension, generalized atherosclerosis, coronary artery disease, history of myocardial infarction, as well as the aneurysm size, localization, type of the procedure and vascular graft material. The variables identified as significant by univariate analysis were selected for multivariate analysis with the Cox proportional hazards regression model to identify the independent predictors. A *p*-value < 0.05 was considered statistically significant.

## 3. Results

### 3.1. Patient Demographics and Descriptive Data

There was no statistically significant difference between patients with true aneurysms and pseudoaneurysms in terms of age. More males were treated for true aneurysm compared to pseudoaneurysm (65 (91.6%) vs. 30 (76.9%); *p* = 0.03).

In the whole group, 90 (81.8%) patients had comorbidities. The most common comorbidity was arterial hypertension, noted in 77 (70%) patients, followed by generalized atherosclerosis (n = 43; 39.1%) and coronary artery disease (n = 29; 26.4%). More patients with pseudoaneurysm had generalized atherosclerosis compared to the true aneurysm group (23 (59%) vs. 20 (28.2%); *p* = 0.002). For the remaining comorbidities and the overall presence of comorbidities, there were no statistically significant differences. History of cigarette smoking was reported by 52 (47.3%) of patients, while 38 (34.5%) patients were currently smoking cigarettes with no significant differences between groups (*p* = 0.8; *p* = 0.5, respectively)

On admission, 71 (64.5%) patients were presenting clinical symptoms. The most common was lower limb pain noted in 43 (39.1%) patients, followed by acute limb ischemia (n = 21; 9.1%) and intermittent claudication (n = 17; 15.5%). Acute limb ischemia was reported more often in the true aneurysm group compared to the pseudoaneurysm group (18 (25.4%) vs. 3 (7.7%); *p* = 0.02). For the remaining clinical symptoms and the overall presence of clinical symptoms, no statistically significant differences were observed.

Fifty-seven (51.8%) patients were diagnosed with other aneurysms (in addition to the treated one), with the most common being the abdominal aorta aneurysm (n = 21; 19.1%). Patients with true aneurysms more often had other aneurysms than patients in the pseudoaneurysm group (42 (59.2%) vs. 15 (38.5%); *p* = 0.04). There was no statistically significant difference between groups in terms of other aneurysm localizations (Table 1).

### 3.2. Characteristics of Aneurysms in the Study Population

The most common etiology of pseudoaneurysm was anastomotic pseudoaneurysm (n = 37; 94.9%). Meanwhile, most true aneurysms had idiopathic etiology (51; 46.4%). The popliteal artery was the most common localization of aneurysms (n = 54; 49.1%), followed by the common femoral artery (n = 53; 48.2%), superficial femoral artery (n = 2; 1.8%), and deep femoral artery (n = 1; 0.9%). In the patients with true aneurysms, significantly more often, the aneurysm was located in the popliteal artery compared to the pseudoaneurysm group (52 (73.2%) vs. 2 (5.1%); *p* < 0.001), while the common femoral artery was more often the localization of the pseudoaneurysm compared to the true aneurysm (36 (92.3%) vs. 17 (23.9%); *p* < 0.001). This finding can be associated with the fact that most of the pseudoaneurysm in our group was a complication of femoral anastomosis (n = 36; 92.3%). Median aneurysm size was 36 IQR 20 mm, with no significant difference between groups (Table 2).

### 3.3. Procedural Characteristics and Outcomes in Patients Undergoing Aneurysm Repair

The median duration of the procedure was 165.5 (45–365) IQR 77.5 min. In 103 (93.7%) patients, intraoperative blood loss was <400 mL. Intraoperative transfusion of red blood cells (RBC) was needed in 6 (5.5%) patients, while intraoperative transfusion of fresh frozen plasma was needed in 2 (1.8%) patients. There was no statistically significant difference in the duration of the procedure or intraoperative blood loss, transfusion of red blood cells (RBC), or transfusion of fresh frozen plasma (FFP) between the two groups.

The most common type of the procedure was aneurysmectomy, which was performed in 73 (66.4%) patients; it was followed by endovascular treatment (n = 17; 15.5%). Aneurysmectomy was performed more often in patients with pseudoaneurysm than with true aneurysm (34 (87.2%) vs. 39 (54.9%); *p* < 0.001), while endovascular treatment and surgical bypass were performed more often in true aneurysm group compared with pseudoaneurysm group (16 (22.5%) vs. 1 (2.6%); *p* = 0.01, 15 (21.1%) vs. 0 (0%); *p* < 0.001). There was no statistically significant difference in the performance of aneurysmectomy with bypass between both groups.

The median duration of hospitalization was 8 (4–100) IQR 4 days. There was no significant difference in the duration of hospitalization and duration of the hospitalization after the procedure between patients with true aneurysms and pseudoaneurysms.

Early postoperative complications occurred in 25 (22.7%) patients; the most common were as follows: surgical site infection (n = 10; 9.1%), hematoma (n = 8; 7.3%), acute limb ischemia (n = 4; 3.6%), myocardial infarction (n = 1; 0.9%), femoral abscess (n = 1; 0.9%), and C.Difficile infection (n = 1; 0.9%). Early postoperative reoperation was needed in 10 (9.1%) patients due to the hematoma (n = 5; 4.5%), acute limb ischemia (n = 4; 3.6%), and femoral abscess (n = 1; 0.9%). There was no significant difference in the overall occurrence of early postoperative complications and in the individual complications, as well as in the performance of early reoperations between both groups. In-hospital mortality was 0% (Table 3).

In univariate logistic regression analysis, the occurrence of postoperative complications was associated only with the occurrence of generalized atherosclerosis in patients (*p* = 0.02, Odds ratio = 3, 95% confidence interval (95% CI) = 1.2–7.7). Therefore, it was the only independent predictive factor for early postoperative complications (Table 4).

### 3.4. Long-Term Follow-Up and Outcomes in Patients Undergoing Aneurysm Repair

The median follow-up time was 16 (1–68) IQR 31 months. There was no statistically significant difference in follow-up time between both groups.

During the follow-up period, a total of 27 (24.5%) late postoperative complications occurred. There was no statistically significant difference between patients with true aneurysm and pseudoaneurysm in the overall occurrence of late postoperative complications and in the individual complications.

Late postoperative reoperations were needed in a total of 13 (11.8%) patients due to the acute limb ischemia (n = 9; 8.2%), prosthesis infection (n = 2; 1.8%), recurrence of pseudoaneurysm (n = 1; 0.9%), and popliteal abscess (n = 1; 0.9%). There were no significant differences between both groups in the performance of late reoperations. In addition, in five (4.5%) patients, due to acute limb ischemia, the recombinant tissue plasminogen activator (r-tPA) therapy was required.

During the follow-up period, three (2.7%) deaths occurred due to acute heart failure (n = 1; 0.9%), intracerebral hemorrhage (n = 1; 0.9%), and traffic accidents (n = 1; 0.9%). There were no significant differences between both groups in terms of mortality (Table 5).

### 3.5. Reoperations

**Figure 3:**
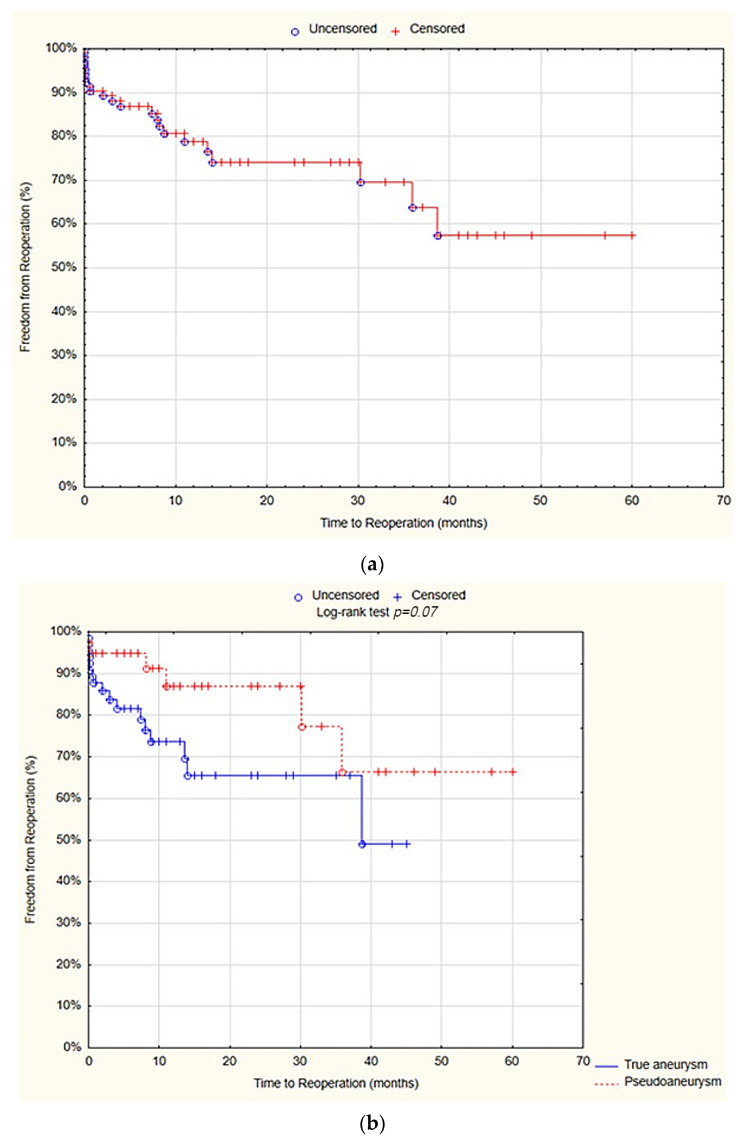
Freedom from reoperations after 12 months—overall (**a**), true aneurysm vs. pseudoaneurysm group (**b**) (Statistica^®^, 13.3, StatSoft).

On survival analysis, the freedom from reoperations at 12 months was 78.8% standard error (SE) 4.6%. There was no statistically significant difference between the true aneurysm and pseudoaneurysm groups in terms of 12 months of freedom from reoperations (73.7% SE 6.3% vs. 87% SE 6.2%; *p* = 0.07; Figure 3).

In univariate Cox proportional hazards regression model analysis, there were no significant differences in freedom from reoperations between patients in terms of age, gender, history of cigarette smoking, current cigarette smoking, presence of comorbidities, aneurysm localization, aneurysm size, the type of the procedure and graft material. Therefore, the multivariate Cox proportional hazards regression model analysis was not performed (Table 6).

### 3.6. Amputations

**Figure 4:**
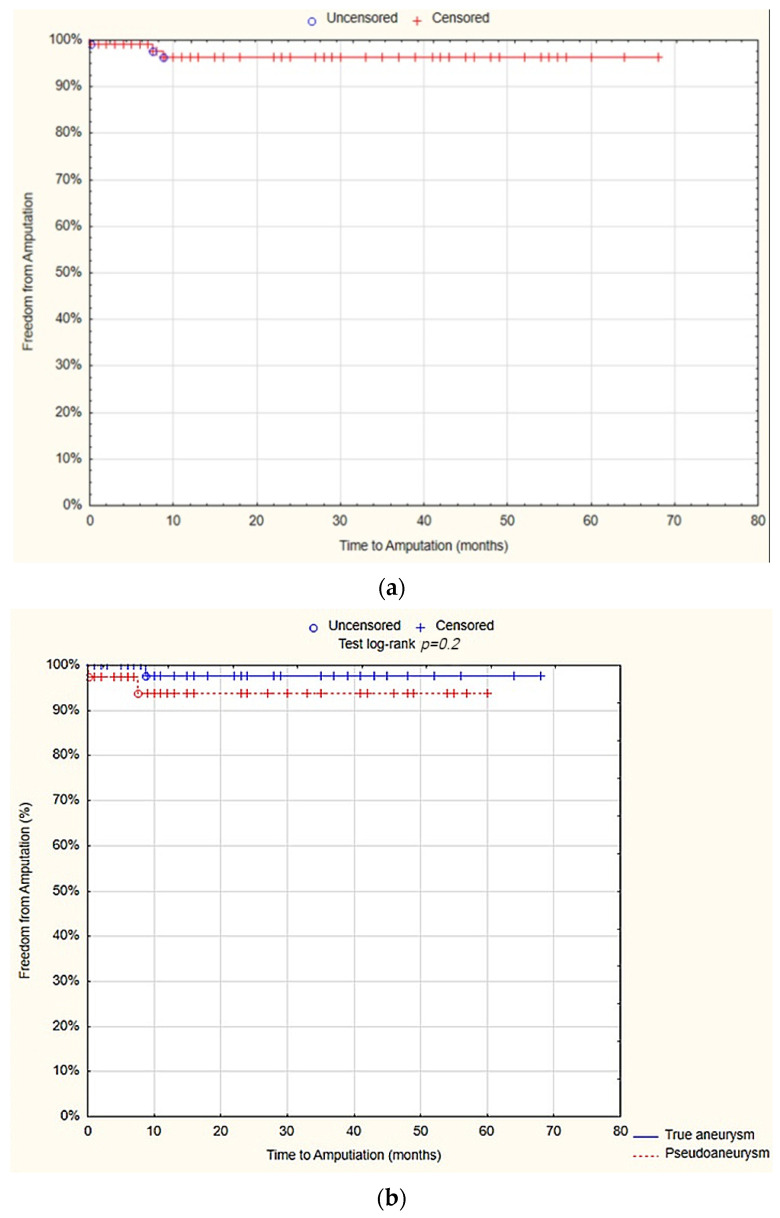
Freedom from amputations after 12 months—overall (**a**), true aneurysm vs. pseudoaneurysm group (**b**) (Statistica^®^, 13.3, StatSoft).

The survival analysis showed that at 12 months, the freedom from amputations after was 96.3% SE 2.1%. There was no statistically significant difference between the true aneurysm and pseudoaneurysm groups in terms of freedom from amputations after 12 months (97.7% SE 2.3% vs. 93.8% SE 4.3%; *p* = 0.2; Figure 4).

No significant differences in freedom from amputations were observed among patients concerning age, gender, history of cigarette smoking, current cigarette smoking, presence of comorbidities, aneurysm localization, aneurysm size, the type of procedure and graft material in the univariate Cox proportional hazards regression model analysis. As a result, multivariate Cox regression model analysis was not conducted (Table 7).

### 3.7. Graft Stenosis

**Figure 5:**
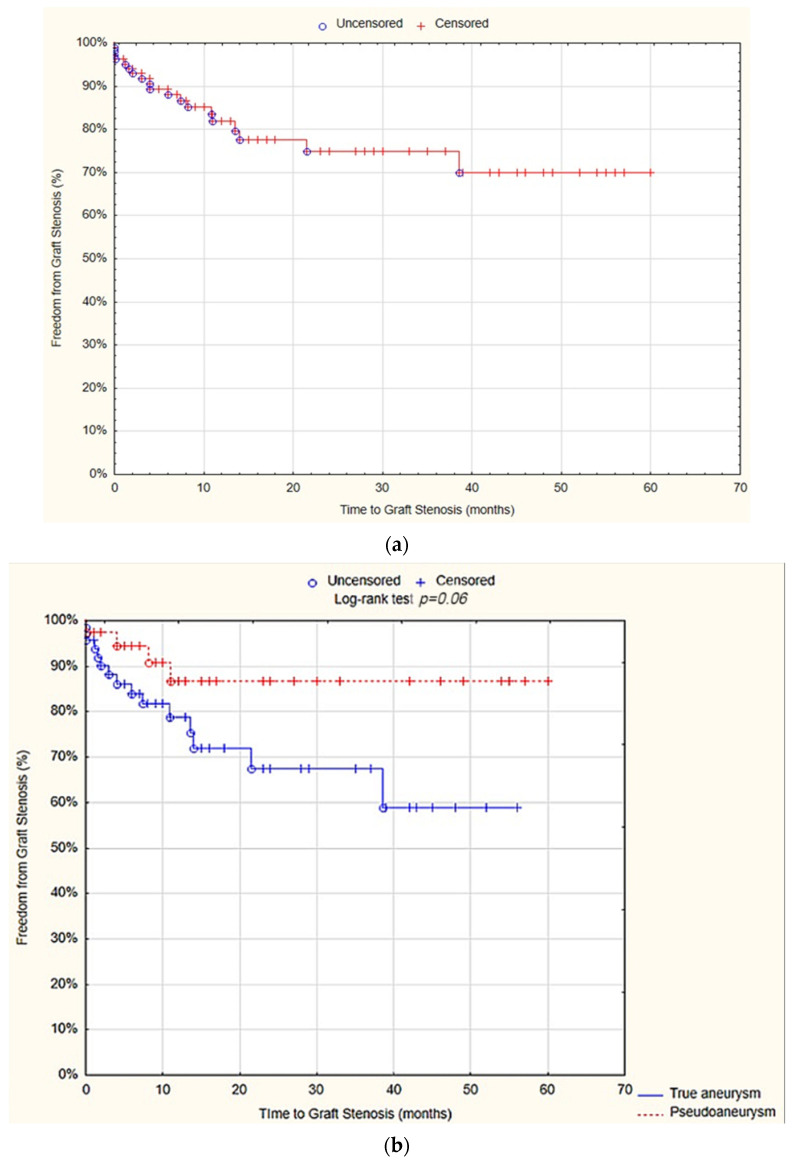
Freedom from graft stenosis after 12 months—overall (**a**), true aneurysm vs. pseudoaneurysm group (**b**) (Statistica^®^, 13.3, StatSoft).

The survival analysis revealed that at 12 months, the freedom from graft stenosis was 81.8% (SE 4.4%). There was no statistically significant difference observed between the true aneurysm and pseudoaneurysm groups in terms of the freedom from graft stenosis after 12 months (78.7% SE 5.9% vs. 86.87% SE 6.3%; *p* = 0.06; Figure 5).

The univariate Cox proportional hazards regression model analysis showed that localization of the aneurysm at the popliteal artery (hazard ratio (HR) = 5.1; 95% CI = 1.8–14.5; *p* = 0.002) and at the common femoral artery (HR = 0.1; 95% CI = 0.04–0.5; *p* = 0.001), as well as the performance of aneurysmectomy (HR = 0.3; 95% CI = 0.1–0.8; *p* = 0.01) and surgical bypass (HR = 3.8; 95%CI = 1.4–10.2; *p* = 0.001), were predictive factors for graft stenosis. However, in the multivariate analysis, none of the above variables proved to be an independent predictor factor (Table 8).

### 3.8. RAS Events

**Figure 6:**
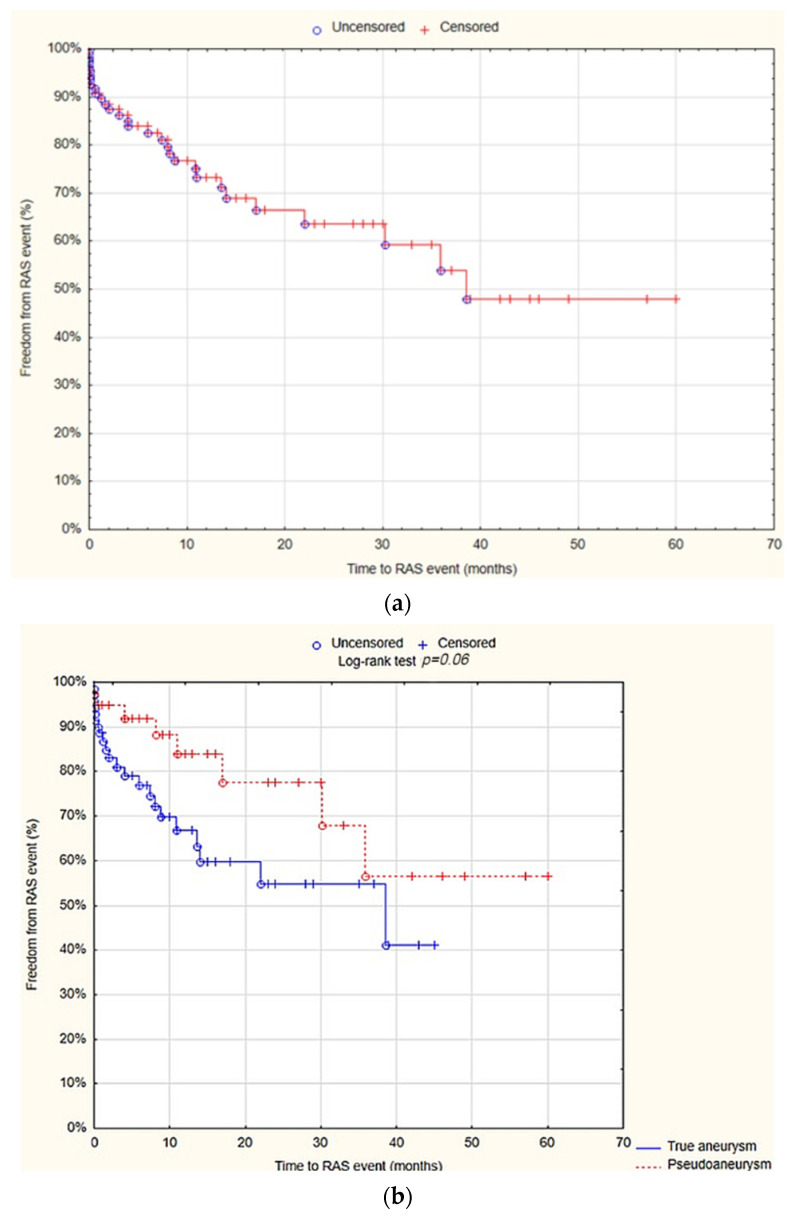
Freedom from reoperation-amputation-stenosis (RAS) events after 12 months—overall (**a**), true aneurysm vs. pseudoaneurysm group (**b**) (Statistica^®^, 13.3, StatSoft). Abbreviations: RAS events—reoperation-amputation-stenosis events.

The survival analysis demonstrated that at 12 months, the freedom from RAS events was 73.3% (SE 5%). There was no statistically significant difference observed between the true aneurysm and pseudoaneurysm groups regarding the freedom from RAS events after 12 months (66.7% SE 6.8% vs. 83.9% SE 6.8%; *p* = 0.06; Figure 6).

The univariate Cox proportional hazards regression model analysis showed that localization of the aneurysm at the popliteal artery (HR= 2; 95% CI = 1–4.3; *p* = 0.04) and at the common femoral artery (HR = 0.4; 95% CI= 0.2–0.9; *p* = 0.002), as well as aneurysm size (HR= 0.3; 95% CI = 0.1–0.8; *p* = 0.01), were predictive factors for RAS events. However, multivariate analysis showed that only the localization of the aneurysm at the common femoral artery (HR = 0.1; 95% CI = 0.02–0.5; *p* = 0.005) and aneurysm size (HR = 1.1; 95% CI 1–1.1; *p* = 0.01) were the independent predictive factors for RAS events (Table 9).

### 3.9. Overall Survival (OS)

**Figure 7:**
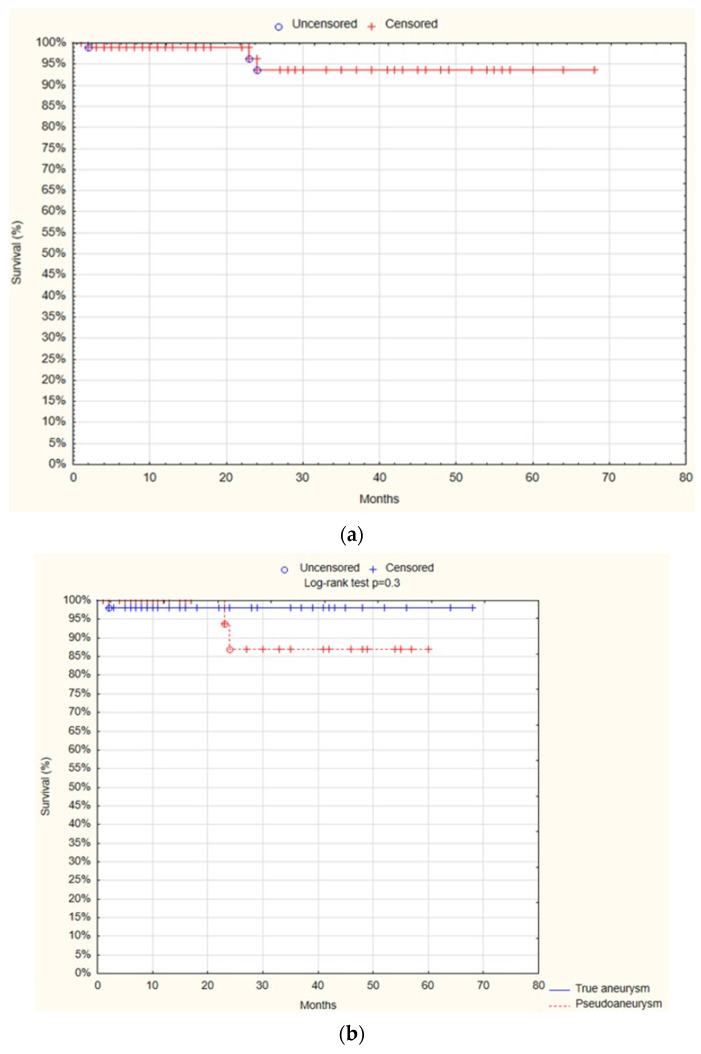
Overall survival—overall (**a**), true aneurysm vs. pseudoaneurysm group (**b**) (Statistica^®^, 13.3, StatSoft).

Finally, the survival analysis demonstrated that at 12 months, OS was 98.2% SE 1%. There was no statistically significant difference observed between the true aneurysm and pseudoaneurysm groups regarding the OS after 12 months (98.2% SE 1.8% vs. 100%; *p* = 0.3; Figure 7).

The univariate Cox proportional hazards regression model analysis showed that only the patient’s age was a positive predictive factor for mortality (HR = 1.2; 95% CI 1–1.3; *p* = 0.01; Table 10).

## 4. Discussion

The study examined demographics, clinical presentations, procedural outcomes, and long-term follow-up of lower limb aneurysm patients. Although age did not differ significantly between true aneurysms and pseudoaneurysms groups, males predominated in the true aneurysms group (91.6% vs. 76.9%, *p* = 0.03). Symptoms included lower limb pain (39.1%), acute limb ischemia (19.1%), and intermittent claudication (15.5%). Acute limb ischemia was more frequent in the true aneurysms group compared to pseudoaneurysm one (25.4% vs. 7.7%, *p* = 0.02). Coexisting aneurysms, notably abdominal aorta aneurysms, occurred in 51.8%, with a higher incidence in the true aneurysms group (59.2% vs. 38.5%, *p* = 0.04). Most aneurysms were located in the popliteal artery (49.1%) or the common femoral artery (48.2%), with pseudoaneurysms mainly due to anastomotic complications in the common femoral artery (92.3%). Aneurysmectomy was the most common procedure (66.4%), especially in the pseudoaneurysms group (87.2%), while endovascular repair predominated in true aneurysms (22.5%). Hospitalization duration and early complications were similar between groups. Long-term outcomes, including complications, reoperations, and mortality, were comparable. Survival analysis indicated comparable outcomes in terms of freedom from reoperations, amputations, graft stenosis, RAS events, and overall survival, with age being the sole predictive factor for mortality.

Lower limb aneurysm epidemiology underscores the male predominance, with true aneurysms occurring nearly twice as often in men compared to women (3% vs. 1.6%) [19]. Sokhal et al. [3] suggest that the female gender may predispose to pseudoaneurysm formation. In our study, more males were treated for true aneurysms compared to pseudoaneurysms (65 (91.6%) vs. 30 (76.9%); *p* = 0.03), whereas more females were treated for pseudoaneurysms than true aneurysms (9 (23.1%) vs. 6 (8.5%); *p* = 0.03). This gender disparity likely arises from multiple factors. Men are frequently exposed to risk factors such as smoking, alcohol use, hypertension, and physical inactivity, which increase the likelihood of developing true arterial aneurysms [20]. Occupational hazards and environmental exposures, common among men in physically demanding or industrial jobs, further contribute to this disparity [21]. Genetic factors also play a role, as familial clustering and specific genetic variations are associated with increased aneurysm risk [22]. Understanding these multifaceted influences—behavioral, hormonal, environmental, and genetic—is crucial for tailoring preventive strategies and personalized treatments for individuals at risk of developing aneurysms [20,21,22].

The incidence of lower limb aneurysms may be underestimated due to a high proportion of clinically silent cases, with the incidence between 30–40% [3,23]. Our study similarly found 39 (35.5%) patients without symptoms. In symptomatic cases, serious complications occurred in 30–77% of patients, including acute limb ischemia and aneurysm rupture [3,4,5,8]. In our group, 47 (66.2%) patients experienced such complications. Acute limb ischemia was more frequent in the true aneurysms group than pseudoaneurysms group (18 (25.4%) vs. 3 (7.7%); *p* = 0.02), possibly linked to pseudoaneurysms primarily arising from arterial anastomosis (37 (94.9%)) Therefore, the patients in this group were on dual antiplatelet therapy, which reduces the risk of acute thrombosis.

Patients with lower limb aneurysms require thorough imaging to detect coexisting aortic, contra-, and ipsilateral aneurysms. Coexisting aneurysm rates range widely (27% to 92%) [3,4,24]. In our study, 57 (51.8%) patients had additional aneurysms, predominantly abdominal aortic aneurysms (AAAs; 21; 19.1%) [3,4,24]. Comprehensive vascular imaging, including bedside ultrasound and Doppler ultrasound, is crucial. Constantino et al. [25] demonstrated 94% to 99% sensitivity of these modalities for AAA [25], and Diwan et al. [2] highlighted the superior sensitivity of the Doppler ultrasound in detecting PAs compared to physical examination [2]. Thus, Doppler ultrasound should be integrated into routine examinations of patients diagnosed with lower limb aneurysms.

Lower limb aneurysms most commonly occur in the popliteal artery and common femoral artery due to their location at the limb’s most mobile joints, which subjects them to unique biomechanical stresses that can weaken arterial walls. The common femoral artery, increasingly used for endovascular procedures, may also be weakened iatrogenically [13]. In our study, the most frequent sites were the popliteal artery (53; 49.1%) and the common femoral artery (52; 48.2%). Pseudoaneurysms predominantly affect the common femoral artery, often associated with catheterization or arterial anastomosis, whereas true aneurysms are more prevalent in the popliteal artery and are often linked with atherosclerosis [4,5,13,26]. Similarly, in our study, pseudoaneurysms were significantly more common in the common femoral artery compared to true aneurysms (36 (92.3% vs. 17 (23.9%); *p* < 0.001), while true aneurysms were more often located in the popliteal artery compared to pseudoaneurysms (52; 73.2% vs. 2; 5.1%; *p* < 0.001).

Surgical management of lower limb aneurysms varies based on etiology, type, and location. Current evidence suggests that pseudoaneurysms resulting from arterial anastomosis are best treated with aneurysmectomy with graft interposition/bypass [27,28,29]. In the study by Markowić et al. [28], 81.6% of patients underwent these procedures, compared to 97.4% in our cohort. The difference possibly occurred due to the absence of infected pseudoaneurysms in our group. Despite advancements in endovascular techniques, their use in lower limb pseudoaneurysms remains limited. Pogorzelski et al. [29] reported only 3.5% of cases treated with endovascular methods, consistent with our finding of 1 (2.6%) patient treated with endovascular methods. Further research is needed to establish the efficacy of endovascular repair in lower limb pseudoaneurysms.

On the other hand, in the treatment of true aneurysms, both open surgical procedures (aneurysmectomy or bypass) and endovascular methods are widely employed. However, recent studies indicate no significant differences in in-hospital mortality, rates of lower extremity amputation, or readmission rates between endovascular methods and open surgery [30,31]. Galiñanes et al. [30] treated 18.5% of patients with endovascular repair and 81.5% with open surgical procedures for true lower limb aneurysms; similarly, in our study, 16 (22.5%) patients underwent endovascular treatment while 55 (77.5%) underwent open surgical procedures.

Early postoperative complications were observed in 25 (22.7%) patients, in a similar number in patients with true aneurysm (16; 22.5%) and pseudoaneurysm (9; 23.1%), with 0% mortality in the whole group. The findings are consistent with the previously published data regarding the management of lower limb aneurysms. In the literature, the postoperative morbidity rate in patients with true aneurysm is 10.2–30%, while mortality is 0–5% [30,31,32]. In the surgical treatment of pseudoaneurysm, morbidity rates range from 21% to 27.6%, while mortality is 0–9.2% [28,29]. The only independent predictive factor for early complications was the occurrence of generalized atherosclerosis (OR = 3, 95% CI = 1.2–7.7, *p* = 0.02). This may be attributed to the fact that generalized atherosclerosis impairs the blood supply to the regions served by the affected artery and additionally elevates the risk of acute thrombosis or infection [33]. In our study, 15 (21.1%) early postoperative complications (10 (9.1%)—Surgical site infection, 4 (3.6%)—Acute limb ischemia, and 1 (0.9%) Myocardial infarction) can be associated with impaired blood supply and/or acute thrombosis and therefore with the occurrence of generalized atherosclerosis.

### Limitations of the Study

The study is primarily constrained by its retrospective design, focusing on a specifically chosen subset of patients from a single medical institution. Moreover, the underrepresentation of females within the study cohort raises concerns regarding the generalizability of findings to the broader female demographic. Additionally, the impact of the COVID-19 pandemic on healthcare access likely led to delayed diagnoses between 2020 and 2022, potentially affecting the presentation of symptomatic aneurysms. Further limitations stem from the inherent biases associated with retrospective analyses. The reliance on available medical records may have introduced selection bias, influencing patient inclusion criteria. Additionally, the study’s single-center approach limits the extrapolation of results to diverse patient populations and healthcare settings, where institutional practices and patient demographics may differ. Finally, the retrospective nature of the study precludes the establishment of causal relationships between variables, while residual confounding factors may have confounded observed associations.

## 5. Conclusions

Symptoms associated with lower limb aneurysms often lack specificity, making their diagnosis challenging and potentially resulting in delayed identification and management. Consequently, patients may present with severe complications endangering life or limb. Predominantly, true aneurysms manifest in the popliteal artery, while pseudoaneurysms, particularly those arising from arterial anastomoses, are typically observed in the common femoral artery. Treatment modalities for lower limb aneurysms include both traditional open surgical approaches and endovascular techniques; nonetheless, the latter warrants further investigation, particularly concerning pseudoaneurysms. Notably, the presence of generalized atherosclerosis may serve as a predictive indicator for the development of early postoperative complications. Given the potential for adverse events, diligent postoperative surveillance is imperative for patients undergoing surgical intervention for lower limb aneurysms. However, it is reassuring that overall rates of postoperative mortality and morbidity remain relatively low.

## Figures and Tables

**Table 1:** Demographic and clinical patient characteristics by type of the aneurysm.

Type of the Aneurysm	True Aneurysm (n = 71; 64.5%)	Pseudoaneurysm (n = 39; 35.5%)	Total (n = 110)	*p*
Age	67 (51–98) IQR 11	68 (15–83) IQR 9	67.5 (15–98) IQR 10	0.9
Gender				0.03
Male	65 (91.6%)	30 (76.9%)	95 (86.4%)	
Female	6 (8.5%)	9 (23.1%)	15 (13.6%)	
Presence of comorbidities	56 (78.8%)	34 (87.2%)	90 (81.8%)	0.3
Arterial hypertension	50 (70.4%)	27 (69.2%)	77 (70%)	0.9
Generalized atherosclerosis	20 (28.2%)	23 (59%)	43 (39.1%)	0.002
Coronary artery disease	16 (22.5%)	13 (33.3%)	29 (26.4%)	0.2
History of myocardial infarction	14 (19.7%)	13 (33.3%)	27 (24.5%)	0.1
Dyslipidemia	11 (15.5%)	8 (20.5%)	19 (17.3%)	0.5
Diabetes mellitus	9 (12.7%)	10 (25.6%)	19 (17.3%)	0.09
Heart failure	5 (7%)	7 (18%)	12 (10.9%)	0.08
COPD	5 (7%)	1 (2.6%)	6 (5.5%)	0.4
Dialysis dependence	4 (5.6%)	0 (0%)	4 (3.6%)	0.3
History of cigarette smoking (yes)	33 (46.48%)	19 (48.72%)	52 (47.3%)	0.8
Current cigarette smoking (yes)	23 (32.39%)	15 (38.46%)	38 (34.5%)	0.5
Clinical symptoms (yes)	47 (66.2%)	24 (61.5%)	71 (64.5%)	0.6
Lower limb pain	27 (38%)	16 (41%)	43 (39.1%)	0.8
Acute limb ischemia	18 (25.4%)	3 (7.7%)	21 (19.1%)	0.02
Intermittent claudication	12 (16.9%)	5 (12.8%)	17 (15.5%)	0.6
Limb swelling	5 (7%)	2 (5.1%)	7 (6.5%)	1
Ulcer	1 (1.4%)	3 (7.7%)	4 (3.6%)	0.1
Aneurysm rupture	2 (2.8%)	1 (2.6%)	3 (2.7%)	1
Gangrene	0 (0%)	2 (5.1%)	2 (1.8%)	0.1
Presence of other aneurysms (yes)	42 (59.2%)	15 (38.5%)	57 (51.8%)	0.04
Abdominal aorta aneurysm	15 (21.1%)	6 (15.4%)	21 (19.1%)	0.5
Popliteal artery aneurysm	15 (21.1%)	3 (7.7%)	18 (16.4%)	0.1
Common femoral artery aneurysm	7 (9.9%)	4 (10.3%)	11 (10%)	1
Superficial femoral artery aneurysm	4 (5.6%)	2 (5.1%)	6 (5.5%)	1
Common iliac artery aneurysm	1 (1.4%)	0 (0.0%)	1 (0.9%)	1

Abbreviations: COPD—chronic obstructive pulmonary disease, IQR—interquartile range.

**Table 2:** Characteristics of aneurysms in the study population.

Type of the Aneurysm	True Aneurysm (n = 71; 64.5%)	Pseudoaneurysm (n = 39; 35.5%)	Total (n = 110)	*p*
Aneurysm localization (artery)				
Popliteal artery	52 (73.2%)	2 (5.1%)	54 (49.1%)	<0.001
Common femoral artery	17 (23.9%)	36 (92.3%)	53 (48.2%)	<0.001
Superficial femoral artery	2 (2.8%)	0 (0%)	2 (1.8%)	1
Deep femoral artery	0 (0%)	1 (2.6%)	1 (0.9%)	0.4
Aneurysm size (mm)	36 IQR 21.5	25 IQR 34.5	36 IQR 20 mm	0.5
True aneurysm etiology				
Idiopathic	51 (81.8%)	-	51 (46.4%)	-
Atherosclerosis	20 (28.2%)	-	20 (18.2%)	-
Pseudoaneurysm etiology				
Arterial anastomosis	37 (94.9%)	37 (38.2%)	-	-
• Aortobifemoral bypass	24 (61.5%)	24 (21.8%)	-	-
• Femoropopliteal bypass	10 (25.6%)	10 (9.1%)	-	-
• Aortofemoral bypass	6 (15.4%)	6 (5.5%)	-	-
• Iliofemoral bypass	2 (5.1%)	2 (1.81%)	-	-
Injury	2 (5.1%)	2 (1.81%)	-	-

Abbreviations: IQR—interquartile range.

**Table 3:** Intraoperative properties and complications.

Type of the Aneurysm	True Aneurysm (n = 71; 64.5%)	Pseudoaneurysm (n = 39; 35.5%)	Total (n = 110)	*p*
Duration of procedure (minutes)	170 (45–315) IQR 97.5	150 (60–365) IQR 79	165.5 (45–365) IQR 77.5	0.2
Type of the procedure				
Aneurysmectomy	39 (54.9%)	34 (87.2%)	73 (66.4%)	<0.001
Endovascular treatment	16 (22.5%)	1 (2.6%)	17 (15.5%)	0.01
Surgical bypass	15 (21.1%)	0 (0%)	15 (13.6%)	<0.001
Aneurysmectomy with bypass	1 (1.4%)	4 (10.3%)	5 (4.5%)	0.05
Intraoperative blood loss				0.4
<400 mL	65 (91.6%)	38 (97.4%)	103 (93.7%)	
>400 mL	6 (4.2%)	1 (2.6%)	7 (5.4%)	
Transfusion of red blood cells (RBC)	5 (7%)	1 (2.6%)	6 (5.5%)	0.4
Transfusion of fresh frozen plasma (FFP)	1 (1.4%)	1 (2.6%)	2 (1.8%)	1
Graft material				0.05
Synthetic	67 (94.37%)	32 (82.1%)	99 (90%)	
Patient’s vein	4 (5.63%)	7 (18)	11 (10%)	
Duration of hospitalization (days)	8 (5–26) IQR 5	9 (4–100) IQR 4	8 (4–100) IQR 4	0.3
Duration of postoperative hospitalization (days)	4 (1–25) IQR 3	5 (2–90) IQR 5	4 (1–90) IQR 4	0.2
Early postoperative complications	16 (22.5%)	9 (23.1%)	25 (22.7%)	1
Surgical site infection	4 (5.6%)	6 (15.4%)	10 (9.1%)	0.2
Hematoma	7 (9.8%)	1 (2.6%)	8 (7.3%)	0.3
Acute limb ischemia	3 (3.2%)	1 (2.6%)	4 (3.6%)	0.7
Myocardial infarction	0 (0%)	1 (2.6%)	1 (0.9%)	0.4
Femoral abscess	1 (1.4%)	0 (0%)	1 (0.9%)	1
C.Difficile infection	1 (1.4%)	0 (0%)	1 (0.9%)	1
Early reoperations	8 (13.8%)	2 (5.7%)	10 (9.1%)	0.3

Abbreviations: C.Difficile—clostridium difficile, IQR—interquartile range.

**Table 4:** Univariate analysis of predictive factors for early postoperative complications after the treatment of lower limb aneurysms using logistic regression.

	Univariable Analysis			
Variable	n (%)	OR	95% CI	*p*
Pre-operative				
Age		1	1–1.1	0.2
Gender				0.7
Male	95 (86.4%)	1		
Female	15 (13.6%)	1.2	0.3–4.7	
Presence of comorbidities	90 (81.8%)	1.8	0.5–7	0.4
Arterial hypertension	77 (70%)	1.1	0.4–3	0.8
Generalized atherosclerosis	43 (39.1%)	3	1.2–7.7	0.02
Coronary artery disease	29 (26.4%)	1.4	0.5–3.8	0.5
History of myocardial infarction	27 (24.5%)	1.6	0.6–4.4	0.3
Dyslipidemia	19 (17.3%)	1.3	0.4–4	0.7
Diabetes mellitus	19 (17.3%)	1.3	0.4–4	0.7
Heart failure	12 (10.9%)	1.8	0.5–6.8	0.4
COPD	6 (5.5%)	3.7	0.7–20	0.1
History of cigarette smoking (yes)	52 (47.3%)	1.6	0.6–3.9	0.3
Current cigarette smoking(yes)	38 (34.5%)	1.08	0.4–2.8	0.8
Aneurysm localization				
Popliteal artery	54 (49.1%)	0.6	0.2–1.5	0.2
Common femoral artery	53 (48.2%)	1.33	0.54–3.3	0.5
Intra-operative				
Type of procedure				
Aneurysmectomy	73 (66.4%)	1.1	0.4–2.9	0.8
Endovascular treatment	17 (15.5%)	1.2	0.4–3.6	0.8
Surgical bypass	15 (13.6%)	0.7	0.2–2.7	0.6
Aneurysmectomy with bypass	5 (4.5%)	2.4	0.4–15	0.4
Blood loss				0.7
>400 mL	7 (6.3%)	1.7	0.1–20.5	
<400 mL				
Duration of procedure		1	0.9–1	1

Abbreviations: COPD—chronic obstructive pulmonary disease, OR—odds ratio, 95% CI—95% confidence interval.

**Table 5:** Follow-up data.

Type of the Aneurysm	True Aneurysm (n = 71; 64.5%)	Pseudoaneurysm (n = 39; 35.5%)	Total (n = 110)	*p*
Follow-up time (months)	16 (1–68) IQR 31	17 (1–60) IQR 33	16 (1–68) IQR 31	0.6
Late postoperative complications	18 (25.4%)	9 (23.1%)	27 (24.5%)	0.7
Acute limb ischemia	14 (19.7%)	3 (7.7%)	17 (15.5%)	0.1
Chronic limb ischemia	3 (4.2%)	1 (2.6%)	4 (3.6%)	1
Popliteal abscess	1 (1.4%)	0 (0.0%)	1 (0.9%)	0.5
Prosthesis infection	0 (0.0%)	2 (5.1%)	2 (1.8%)	0.1
Myocardial infarction	0 (0.0%)	1 (2.6%)	1 (0.9%)	0.4
Recurrence of pseudoaneurysm	0 (0.0%)	1 (2.6%)	1 (0.9%)	0.4
Intracerebral hemorrhage	0 (0.0%)	1 (2.6%)	1 (0.9%)	0.4
Late reoperations	10 (14.1%)	3 (7.7%)	13 (11.8%)	0.1
Mortality	1 (1.4%)	2 (5.1%)	3 (2.7%)	0.3

Abbreviations: IQR—interquartile range.

**Table 6:** Univariate analysis of predictive factors for freedom from reoperations using Cox proportional hazards regression model.

	Univariable Analysis			
Variable	Freedom from Reoperations (Months)	HR	95% CI	*p*
Age		1	1–1.1	0.3
Gender				0.1
Male	8 IQR 14.5	0.5	0.2–1.3	
Female	7.4 IQR 15	1		
History of cigarette smoking				0.6
Yes	7.5 IQR 12.5	0.8	0.4–1.9	
No	8.9 IQR 21.8	1		
Current cigarette smoking				0.2
Yes	8 IQR 14	0.5	0.2–1.4	
No	8.1 IQR 15	1		
Presence of comorbidities				
Arterial hypertension				0.3
Yes	8 IQR 13	1.7	0.6–4.6	
No	8 IQR 22	1		
Generalized atherosclerosis				0.2
Yes	7 IQR 14.5	1.7	0.7–3.8	
No	8.8 IQR 14.5	1		
Coronary artery disease				0.5
Yes	11 IQR 17	0.7	0.3–1.9	
No	8 IQR 14	1		
History of myocardial infarction				0.5
Yes	6 IQR 10.5	0.7	0.2–2.1	
No	8.2 IQR 15.5	1		
Localization				
Popliteal artery	3 IQR 11.3	1.7	0.7–3.8	0.2
Common femoral artery	10 IQR 20.5	0.5	0.2–1.3	0.2
Superficial femoral artery	9.9 IQR 1.1	2.3	0.3–17.4	0.4
Deep femoral artery	12	1		
Aneurysm size		1.1	1–1.1	0.06
Type of the procedure				
Aneurysmectomy	8 IQR 17	0.6	0.3–1.4	0.3
Endovascular treatment	4 IQR 28	1.5	0.6–4.2	0.4
Bypass	8 IQR 12.8	2.06	0.8–5.6	0.2
Aneurysmectomy with bypass	12	1		
Graft material				
Synthetic	11 IQR 19.5	1		
Patient’s vein	3 IQR 22.95	4.64	0.51–42.56	0.17

Abbreviations: HR—hazard ratio, 95% CI—95% confidence interval, IQR—interquartile range.

**Table 7:** Univariate analysis of factors with freedom from amputations using Cox proportional hazards regression model.

	Univariable Analysis			
Variable	Freedom from Amputations (Months)	HR	95% CI	*p*
Age		1	0.9–1.1	0.9
Gender				0.3
Male	11 IQR 29.5	0.3	0.02–3.3	
Female	10 IQR 23.5	1		
History of cigarette smoking				0.7
Yes	10 IQR 17.5	0.6	0.1–6.5	
No	15 IQR 36.5	1		
Current cigarette smoking				1
Yes	10 IQR 17.5	1	0.1–11	
No	11 IQR 32.8	1		
Presence of comorbidities				
Arterial hypertension				0.8
Yes	11 IQR 25	0.7	0.1–8.3	
No	9 IQR 32	1		
Generalized atherosclerosis				0.3
Yes	11 IQR 31.5	3.2	0.3–35.7	
No	11 IQR 27	1		
Coronary artery disease				1
Yes	13 IQR 27	1		
No	10 IQR 28	1		
History of myocardial infarction				1
Yes	11 IQR 21	1		
No	11 IQR 30.5	1		
Localization				
Popliteal artery	9 IQR 24	1		
Common femoral artery	13 IQR 32	2.2	0.2–24	0.5
Superficial femoral artery	9.9 IQR 1.1	1		
Deep femoral artery	12	1		
Aneurysm size		1	1–1.1	0.2
Type of the procedure				
Aneurysmectomy	10 IQR 23	1.1	0.1–12.1	0.9
Endovascular treatment	22 IQR 37	2.9	0.3–32.1	0.4
Bypass	15 IQR 20	1		
Aneurysmectomy with bypass	12	1		
Graft material				
Synthetic	11 IQR 29.8	1		
Patient’s vein	3 IQR 22.3	6.4	0.6–73.2	0.6

Abbreviations: HR—hazard ratio, 95% CI—95% confidence interval.

**Table 8:** Uni- and multivariate analysis of factors with freedom from graft stenosis using Cox proportional hazards regression model.

	Univariable Analysis				Multivariable Analysis		
Variable	Freedom from Graft Stenosis (Months)	HR	95% CI	*p*	HR	95% CI	*p*
Age		1	1–1.1	0.7			
Gender				0.2			
Male	10 IQR 20.3	0.5	0.2–1.5				
Female	7.4 IQR 15	1					
History of cigarette smoking				0.4			
Yes	9.5 IQR 12.5	0.7	0.3–1.7				
No	9.5 IQR 21.8	1					
Current cigarette smoking				0.2			
Yes	10 IQR 13.5	0.5	0.2–1.5				
No	9 IQR 21	1					
Presence of comorbidities							
Arterial hypertension				0.8			
Yes	10 IQR 16	0.9	0.3–2.3				
No	8 IQR 22	1					
Generalized atherosclerosis				0.4			
Yes	10 IQR 16	0.6	0.2–1.7				
No	9 IQR 20.5	1					
Coronary artery disease				0.2			
Yes	11 IQR 22	0.4	0.1–1.5				
No	9 IQR 14.4	1					
History of myocardial infarction				0.2			
Yes	10 IQR 15.8	0.4					
No	9 IQR 21	1					
Localization							
Popliteal artery	5 IQR 11.3	5.1	1.8–14.5	0.002	1.2	0.2–1.4	0.8
Common femoral artery	13 IQR 27	0.1	0.04–0.5	0.001	0.2	0.03–1.4	0.1
Superficial femoral artery	26.5 IQR 15.5	1					
Deep femoral artery	12	1					
Aneurysm size		1	1–1.1	0.3			
Type of the aneurysm							
True aneurysm	8 IQR 14.5	1					
Pseudoaneurysm	11 IQR 19	0.4	0.1–1.1	0.08			
Type of the procedure							
Aneurysmectomy	10 IQR 19	0.3	0.1–0.8	0.01	0.8	0.2–2.5	0.7
Endovascular treatment	4 IQR 36	2.2	0.8–6	0.1			
Bypass	6 IQR 12.8	3.8	1.4–10.2	0.001	1.7	0.2–6	0.4
Aneurysmectomy with bypass	11 IQR 1	1					
Graft material							
Synthetic	10 IQR 18.4	1					
Patient’s vein	3 IQR 22.3	0.6	0.1–4.5	0.6			

Abbreviations: HR—hazard ratio, 95% CI—95% confidence interval.

**Table 9:** Uni- and multivariate analysis of factors with freedom from RAS events using Cox proportional hazards regression model.

	Univariable Analysis				Multivariable Analysis		
Variable	Freedom from RAS Events (Months)	HR	95% CI	*p*	HR	95% CI	*p*
Age		1	1–1.1	0.5			
Gender				0.4			
Male	8.8 IQR 15	0.7	0.25–1.8				
Female	7.4 IQR 22	1					
History of cigarette smoking				0.9			
Yes	8.1 IQR 14	1.1	0.5–2.1				
No	8.4 IQR 21.4	1					
Current cigarette smoking				0.5			
Yes	8.5 IQR 16	0.8	0.4–1.7				
No	8.1 IQR 14.2	1					
Presence of comorbidities							
Arterial hypertension				1			
Yes	9 IQR 13	1.1	0.5–2.2				
No	7 IQR 22	1					
Generalized atherosclerosis				0.4			
Yes	7 IQR 15	1.4	0.7–2.8				
No	9 IQR 14.4	1					
Coronary artery disease				0.6			
Yes	11 IQR 14	0.8	0.3–1.9				
No	8 IQR 14	1					
History of myocardial infarction				0.8			
Yes	6 IQR 11	0.9	0.4–2.2				
No	8.8 IQR 15.8	1					
Localization							
Popliteal artery	4 IQR 12.5	2	1–4.3	0.04	0.24	0.1–1.1	0.06
Common femoral artery	10 IQR 22	0.4	0.2–0.9	0.02	0.1	0.02–0.5	0.005
Superficial femoral artery	9.9 IQR 1.1	1.8	0.2–13.4	0.6			
Deep femoral artery	12	1					
Aneurysm size	1.1	1–1.1	0.01	1.1	1–1.1	0.01	
Type of the aneurysm							
True aneurysm	6 IQR 13	1					
Pseudoaneurysm	11 IQR 22	0.5	0.2–1.1	0.07			
Type of the procedure							
Aneurysmectomy	8.2 IQR 16	0.6	0.3–1.3	0.2			
Endovascular treatment	4 IQR 28	1.6	0.7–3.8	0.3			
Bypass	6 IQR 13	2	0.8–4.9	0.1			
Aneurysmectomy with bypass	11 IQR 1	1		1			
Graft material							
Synthetic	8.9 IQR 13.3	1					
Patient’s vein	3 IQR 22.3	0.3	0.04–2.5	0.3			

Abbreviations: HR—hazard ratio, 95% CI—95% confidence interval.

**Table 10:** Univariate analysis of factors with overall survival using Cox proportional hazards regression model.

	Univariable Analysis			
Variable	Survival Time (Months)	HR	95% CI	*p*
Age		1.2	1–1.3	0.01
Gender				0.4
Male	11 IQR 31	0.3	0.03–3.7	
Female	10 IQR 25	1		
History of cigarette smoking				1
Yes	10 IQR 18	1		
No	15.5 IQR 37	1		
Current cigarette smoking				1
Yes	10 IQR 19	1		
No	13.5 IQR 34	1		
Presence of comorbidities				
Arterial hypertension				0.2
Yes	11 IQR 29	0.2	0.02–2.5	
No	15 IQR 32	1		
Generalized atherosclerosis				0.8
Yes	11 IQR 33	0.76	0.07–8.4	
No	11 IQR 31	1		
Coronary artery disease				0.9
Yes	13 IQR 27	1.2	0.1–13.1	
No	10 IQR 33	1		
History of myocardial infarction				0.6
Yes	11 IQR 24	1.8	0.2–20.4	
No	13 IQR 30	1		
Localization				
Popliteal artery	9 IQR 27	0.8	0.07–9.1	0.9
Common femoral artery	15 IQR 32	1.8	0.2–19.8	0.6
Superficial femoral artery	26.5 IQR 15.5	1		
Deep femoral artery	12	1		
Aneurysm size		0.8	0.2–3.4	0.7
Type of the aneurysm				
True aneurysm	10 IQR 33	1		
Pseudoaneurysm	13 IQR 28	3	0.3–33.4	0.4
Type of the procedure				
Aneurysmectomy	10 IQR 23	1	0.1–11.4	1
Endovascular treatment	29 IQR 37	1.9	0.2–21.7	0.6
Bypass	15 IQR 32	1		1
Aneurysmectomy with bypass	11 IQR 1	1		1
Graft material				
Synthetic	11.5 IQR 31.3	1		
Patient’s vein	5.5 IQR 22.3	1		1

Abbreviations: HR—hazard ratio, 95% CI—95% confidence interval.

## Data Availability

The raw data supporting the conclusions of this article will be made available by the authors on request.
